# Translation, Cultural Adaptation and Preliminary Psychometric Testing of the Managerial Ethical Profile Scale for Finnish Health and Social Care Sector

**DOI:** 10.1111/scs.70101

**Published:** 2025-08-31

**Authors:** Essi Xiong, Maarit Karhula, Jaana Paltamaa, Hannu Vähänikkilä, Niko Männikkö, Gian Luca Casali, Outi Kanste

**Affiliations:** ^1^ Faculty of Medicine, Research Unit of Health Sciences and Technology University of Oulu Oulu Finland; ^2^ Research Unit The Social Insurance Institution of Finland Helsinki Finland; ^3^ School of Health and Social Studies Jamk University of Applied Sciences Jyväskylä Finland; ^4^ Faculty of Medicine, Northern Finland Birth Cohorts, Arctic Biobank, Infrastructure for Population Studies University of Oulu Oulu Finland; ^5^ Centre for Research and Innovation Oulu University of Applied Sciences Oulu Finland; ^6^ Faculty of Business and law, School of Management Queensland University of Technology (QUT) Brisbane Queensland Australia; ^7^ Medical Research Center Oulu, Oulu University Hospital University of Oulu Oulu Finland

**Keywords:** cross‐sectional survey, cultural adaptation, ethical decision‐making, ISPOR protocol, management, translation

## Abstract

**Background:**

The Managerial Ethical Profile (MEP) scale was developed to measure the perceived impact of different elements of common ethical frameworks on managerial ethical decision‐making.

**Aim:**

To translate, culturally adapt and perform preliminary psychometric testing of the MEP scale in the Finnish health and social care sector.

**Design:**

The MEP scale was translated and culturally adapted according to the International Society for Pharmacoeconomics and Outcomes Research (ISPOR) protocol based on a cross‐sectional online survey.

**Participants:**

Finnish health and social care managers and experts.

**Ethical Consideration:**

Research permits were obtained from all participating organisations in accordance with the organisations' guidelines. Participation was voluntary and participants gave informed consent.

**Methods:**

Translation and cultural adaptation of the Finnish version of the MEP scale (MEP‐Fin) was performed and perceived relevance and content validity were assessed. An online survey was distributed to homecare service managers in 2022, and a total of 68 managers participated. The data were analysed using descriptive statistics (means, standard deviations, Pearson's correlation coefficients and Cronbach's alphas).

**Results:**

The translation, cultural adaptation and preliminary psychometric testing process identified issues regarding the understanding of the MEP scale in the Finnish context. The MEP‐Fin showed high overall content validity (S‐CVI/Ave 0.91). The mean score for the comprehensibility of the scale was 8.4. Content validity was high for all individual items (I‐CVI range: 0.56 to 1.00), except for one item (0.38). The Cronbach's alphas for the eight subscales were between 0.51 and 0.93, and for the whole scale, 0.84.

**Conclusions:**

The systematic translation and cultural adaptation process resulted in a conceptually equal MEP‐Fin, which was perceived as thorough and relevant. It showed good coverage, content validity and internal consistency. Preliminary psychometric testing provided information on the characteristics of the MEP‐Fin for validation. Further studies with larger data sets and different cultures are needed.

## 1 Introduction

In the 2020s health and social care management has been under unprecedented pressure globally. The COVID‐19 pandemic, labour shortages, the aging population and advances in technology have brought complex ethical decision‐making (EDM) under intense scrutiny [1, 2, 3]. Studies have found EDM to be challenging [4, 5] highlighting the importance of understanding the person and the organisation behind the EDM [5, 6]. Analysis of cross‐cultural effects and the development of complex hypotheses and new methodologies have been proposed [4] for EDM studies.

Understanding and measurement of EDM in the health and social care sector has mostly focused on either health care [5] or social care [7] in isolation. Some studies have looked at health and social care in an integrated way, but without considering EDM [8]. The use of ethical frameworks increases ethical awareness and reduces moral disengagement in practice [9] and is paramount to the success of any organisation [6]. Ethical leadership in health and social care organisations has been shown to have positive effects on patients, staff and managers [5, 7]. In addition to providing quality care, it also contributes to the achievement of organisational objectives and efficacy [10]. There are few validated measures of EDM by health and social care managers. There is a clear need for valid, reliable and accessible measurement of EDM in health and social care management in Finland, as no scales have been available in Finnish to date.

## 2 Background

In management, EDM is seen as a dynamic and complicated process involving individual themes and organisational factors [4]. In addition, EDM is seen as a collective and situational process, expressed through interaction [9]. External factors such as legal, political, economic and competitive considerations can have a significant impact on ethical decision‐making by managers [3]. Various models and theories have been developed to understand it, but empirical testing has been limited [3, 11, 12]. The challenges and complexity of EDM have caused frustration among researchers [13]. The validity of existing EDM measures has been questioned [14] and impacts on daily practices are poorly understood [5]. By identifying, making problems visible and assessing the factors that influence ethical decision‐making by managers, it is possible to tailor appropriate policies and training for the organisation [5, 15].

In research, moral sensitivity is usually associated with ethicality and commitment to an ethical decision‐making process and prevention of moral abuses [16]. Various factors (gender, age, and education) that influence EDM have been studied [6] and more complex and nuanced relationships have been investigated in studies that reflect the maturation of the research environment [4]. Theoretical models have been developed; the Integrated Ethical Decision‐Making (I‐EDM) model, for example, was designed to especially apply to business contexts [13]. The study by Casali and Perano (2023) analysed data mainly from publications in the business context [6].

Studies on EDM in healthcare have focused on clinical decision‐making situations in nursing and medicine [2, 16, 17, 18]. In the context of rehabilitation, EDM has been examined in person‐centred rehabilitation [19] and in terms of clinical reasoning processes [20]. The ethics of health and social care management have been examined from perspectives such as ethical dilemmas [12], ethical sensitivity [16] and EDM confidence [3]. There is insufficient evidence of the influence of different factors on EDM by managers [6]. Theoretical and empirical studies targeting EDM in healthcare management are sparse, and related quantitative studies are essential [21].

Although several employed instruments such as the Ethical Decision‐Making Measure (EDM) [14] and Measure of Managerial Moral Judgement (MMMJ) [22] have been developed within business‐oriented frameworks and validated primarily among students and executive cohorts, the MEP scale demonstrates significantly greater contextual alignment and multidimensional relevance within healthcare management [15] (Table S1). Its psychometric validation among managers in health care settings [15] along with preliminary applications in Nordic social and/or health care settings [23, 24] reinforces both its construct validity and its methodological relevance to the present study. In the process of evaluating potential instruments, the Ethical Decision‐Making Confidence Scale for Nurse Managers (EDMC), developed by Birkholz et al. (2022), was also examined [3]. As this study sought an instrument grounded in foundational ethical theory to facilitate a more comprehensive and conceptually coherent understanding of ethical decision‐making, alternative measures were deemed to be aligned with these objectives.

The MEP scale measures the ethical profiles of managers and captures widely their ethical preferences (Table S1). The MEP scale consists of items which represent and measure divergent principles from four main schools of moral philosophy: economic egoism, reputational egoism, virtue ethics and rule deontology. To measure ethical preferences, the MEP scale uses five‐point Likert response options: extremely important [5], very important [4], fairly important [3], not very important [2] and not important at all [1]. The original English version of the MEP scale has been psychometrically tested. Based on confirmatory factor analysis, the statistics for the 21‐item ‘best fit model’ are deemed good or adequate [25]. The eight‐factor structure demonstrated an acceptable model fit (CFI = 0.933, RMSEA = 0.057, SRMR = 0.0467), despite a statistically significant chi‐square value (*χ* ^2^ = 191.60, df = 161, *p* < 0.001). These results provide support for the construct validity of the SD model [15]. It consists of eight subscales that accurately measure ethical constructs related to ethical decision‐making [15, 25] which are described in Table S2.

In addition to Australia, the use of the MEP scale in health care has been studied in Sweden, where it has been used to measure distributed leadership [24] and to measure gender equality in services for older people and social work [23]. Previous studies in Sweden only partially used the MEP scale and did not look at translation and cultural adaptation in depth. In the Czech Republic, the 24‐item MEP scale was used to examine social work managers' disposition to EDM [7] but this study did not report on the process of translating the measure or cultural adaptation. To our knowledge, no Finnish studies have used the MEP scale, and cultural validation has not been systematically carried out for other languages either. Systematic translation and cultural adaptation of the MEP‐Fin is necessary [26, 27] so that it can be used in different linguistic and cultural contexts.

### 2.1 Aim

To translate, culturally adapt and perform preliminary psychometric testing of the MEP scale in Finnish health and social care.

## 3 Methods

### 3.1 Study Design

Translation and cultural adaptation of the original 24‐item MEP scale was performed according to the ISPOR protocol. A cross‐sectional online survey was distributed to homecare service managers. Reporting of the study complied with the COnsensus‐based Standards for the selection of health status Measurement INstruments (COSMIN) checklist [28] (Supporting Information).

### 3.2 Preparation

**FIGURE 1:**
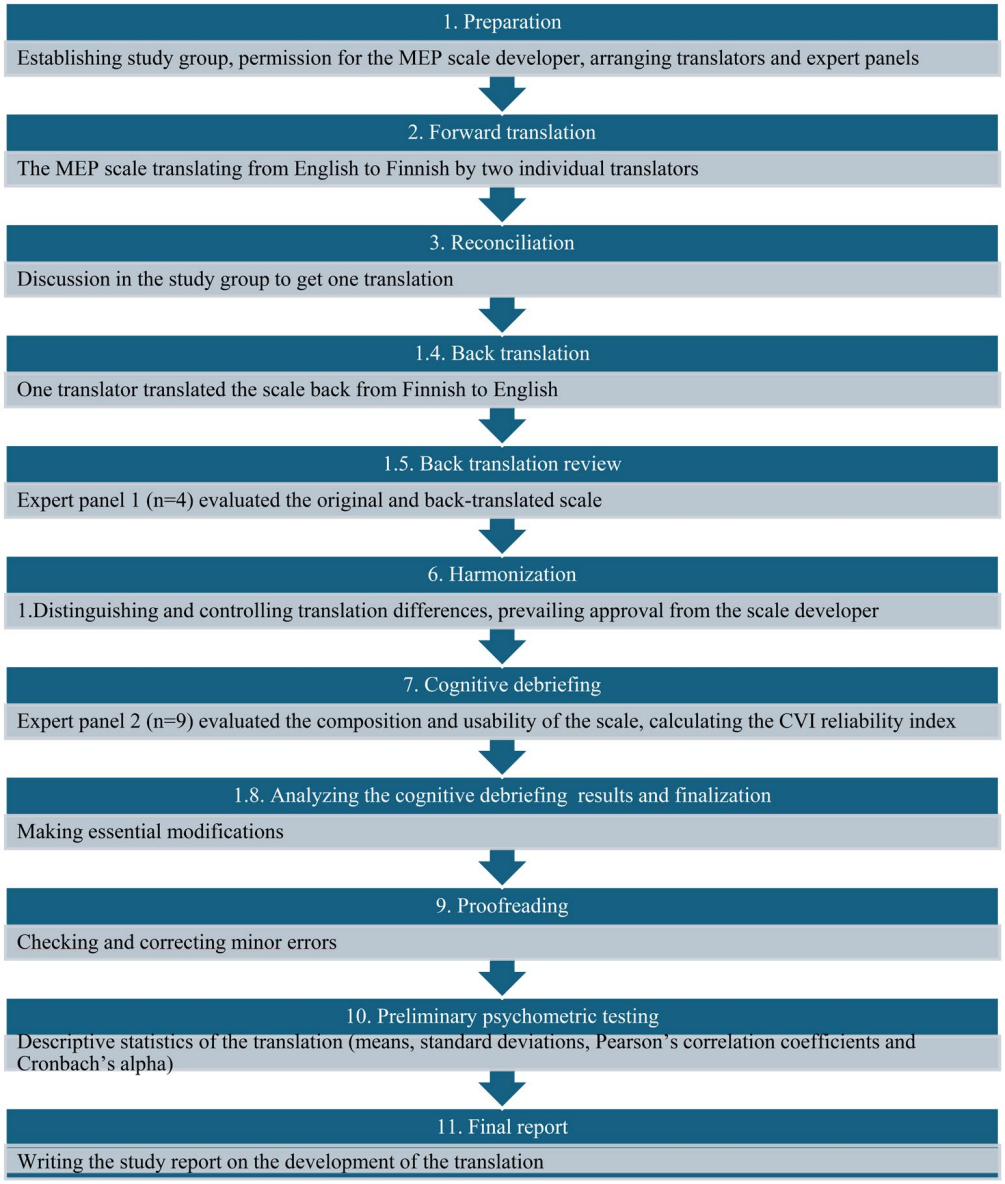
The transition and cultural adaptation of the MEP‐fin parallel to ISPOR guidelines (Wild et al. 2005) and added preliminary psychometric testing phase. MEP‐fin, Finnish version of the MEP scale; The MEP scale, The Managerial Ethical Profile scale.

A study group was established, whose members discussed conceptual and semantic revisions until consensus on each translation draft was reached, and the MEP‐Fin scale was deemed culturally usable. Permission to translate the MEP scale was obtained from the scale developer Luca Gian Casali. The cooperation agreement was concluded with three certified translators. Health and social care managers and experts were recruited to participate in the study. Two different expert panels (1 and 2) were formed and used in various phases of the process. Translation and cultural adaptation were carried out in 2022. The ISPOR guidelines provide a systematic process including ten specific phases [26, 27] (Figure 1). This ensures a comprehensive understanding of the scale's properties, characteristics and underlying assumptions. This study looks at the 24‐item scale, as did a previous study in a European context [7] although Casali omitted three of these items in the originally published scale (‘Attaining organizational yearly budgets/short term’, ‘Generating the greatest overall benefits for the district/hospital’ and ‘Obeying the law/state and federal’) due to cross‐loadings [15] (Table S1).

### 3.3 Forward Translation

Two native Finnish‐speaking certified English translators independently translated the items, instructions and response options of the MEP scale, resulting in two Finnish translations (T1 and T2) [26].

### 3.4 Reconciliation

Translations T1 and T2 were compared, and any inconsistencies were discussed and resolved among the study group. After consensus on idioms and preferred phrasing was reached, the two translations were merged into a single forward translation that was used for back translation. The construction of a single forward translation resulted in a scale from which misunderstandings were eliminated as carefully as possible.

### 3.5 Back Translation

The MEP‐Fin was back translated by a native English‐speaking certified translator, as recommended [25]. This translator was not familiar with the original English version. This phase enhanced the quality of the scale development as the study group was able to evaluate whether any of the nuances of the original scale had been lost in the back translation process [26, 27].

### 3.6 Back Translation Review

The back translation was compared to the original English version for conceptual equivalence by experts and the study group. Expert Panel 1 (*n* = 4) consisted of health and/or social care managers. The inclusion criteria for the panellists were a minimum of 10 years of management experience in health and/or social care, as well as possession of at least a master's degree. Managers had Finnish as their mother tongue and, in addition, sufficient English language skills, which they had acquired during their training and that they judged themselves to be sufficient to participate in the translation work. Expert Panel 1 evaluated translations using a form which presented the original and translated items in parallel. For each item, the experts were asked to answer the question ‘Does the translated version capture the content of the original?’ with either ‘yes’ or ‘no’. The experts were also given the opportunity to write open‐ended answers, which were documented by the researcher during the expert panel meeting to increase clarity.

### 3.7 Harmonisation

The study group examined the results of the Expert Panel 1 discussions to identify and target differences between the two translations. The back translation was compared with the original instrument to ensure the overall meanings of the scale items were retained. Any discrepancies between the original English version and the back translation were discussed among the study group, and alternate translations were proposed which, while not literal, were considered to capture the content of the original better.

These discussions generated one combined forward translation. After harmonisation, approval for the back translation was sought from the scale developer. He approved the back‐translated version of the MEP scale.

### 3.8 Cognitive Debriefing

Cognitive debriefing interviews with the members of Expert Panel 2 (*n* = 9; four health and social care managers and five health and social care research experts) were conducted, in addition to pretesting using self‐administered questionnaires. The inclusion criteria for the panellists were management experience of a minimum of 10 years in health and/or social care, as well as possession of at least a master's degree. Experts had relevant research experience and an academic background. Expert Panel 2 assessed the MEP scale items using a four‐point scale: not relevant at all [1], somewhat relevant [2], quite relevant [3] and highly relevant [4].

The cognitive debriefing interviews aimed to find out whether participants understood the meaning of items, instructions and response options, and assessed the comprehensiveness of the content and the cultural relevance of the MEP‐Fin. One researcher conducted the interviews online through Microsoft Teams. All interviews were recorded with the permission of participants, allowing the researcher to return the data later. The interviews were guided by a cognitive interview guide. Experts were asked to use the ‘think aloud’ method, that is, to verbalise what they thought of each item, instruction, and response option [29]. Any questions, instructions or response options identified by a participant as difficult to understand were discussed between the participant and the researcher. The participant was asked to provide an alternative word or phrase, if necessary, that would make the item easier to understand.

Questions were also asked about content and cultural appropriateness, and whether any content relevant to the phenomenon was missing. Difficulties identified by participants and suggested changes were documented by the researcher on a worksheet during the meeting. The experts were also encouraged to write remarks on this document. Participants were given time to go through the analysis and send a written evaluation document within a few weeks. All the collected material was discussed later among the study group members and with the scale developer. Necessary changes identified in the cognitive debriefing interviews were integrated into the MEP‐Fin. A review of cognitive debriefing results and comparison with the original version of the scale is key to convincing cultural applicability [26].

### 3.9 Cognitive Debriefing Results, Proofreading and Finalisation

Based on the results of the evaluation by Expert Panel 2, an agreed list of proposed changes to the scale was drawn up. The Content Validity Index (CVI) score of the MEP‐Fin was calculated, as this is the most commonly used method of calculating content validity quantitatively. Two CVI scores were calculated: Item‐CVI (I‐CVI) and Scale‐level CVI (S‐CVI). For satisfactory validity, the I‐CVI score should exceed 0.78, and the S‐CVI/Ave should be 0.90 or higher if there are more than six experts [30]. In this study, the experts also rated the validity of the entire scale from 1 to 10. All the cultural adaptation procedures are traceable through the appropriate documentation. Some changes were necessary because there are no similar sayings or terms in the Finnish language. For this reason, the concepts of certain items had to be clarified and explained in more detail. No conceptually problematic items were found that could not be overcome.

Any spelling and grammatical errors in the final translation were corrected. Quality assurance was conducted by the study group to ensure that any possible minor errors on the scale were corrected. Changes or rewordings were discussed among the research group, considering the cognitive debriefing interviews and further discussed with the scale developer. This phase involved more advanced translation and cultural adaptation to further develop the MEP‐Fin for preliminary psychometric testing [27].

### 3.10 Preliminary Psychometric Testing

The data were collected in autumn 2022 using a cross‐sectional online survey completed by homecare service managers in five public health and social care authorities and fifteen municipalities or cities across Finland. The inclusion criteria for the participants were to work as a manager and that all managers in the target organisations were middle/top or frontline managers or responsible for services for older people at homes and sufficient language skills to answer the questionnaire in Finnish or Swedish, which are the official languages in Finland. The online survey was sent to 288 managers, and 68 answered (30% response rate). There were no missing values in the survey data. The questionnaire included background variables (gender, educational level, job description, age, time allocated to management work and years in current position) and the MEP‐Fin.

The data were analysed using descriptive statistics (means, standard deviations, Pearson's correlation coefficients and Cronbach's alphas). Perceived relevance and content validity were assessed using descriptive statistical methods. The data were analysed with IBM SPSS Statistics (version 28.0) [31].

The sum variables were formed according to the original 21‐item ‘best fit’ model structure. Internal consistency was evaluated using Cronbach's alpha. The accepted rule‐of‐thumb is that the alpha value should be at least 0.70 for a scale to have an acceptable level of internal consistency, although there are limited grounds for endorsing such a heuristic [32]. The relationships between the variables were calculated with Pearson's correlation coefficients. The correlation coefficients were determined to be weak if the absolute value of the correlation coefficient was less than 0.39, moderate if the value was 0.40–0.69, strong if the value was 0.70–0.89 and very strong if the value was 0.90–1.00 [33].

## 4 Ethical Considerations

The study followed the Finnish Advisory Board on Research Integrity's ethical guidelines [34] and complied with EU legislation on personal data [35]. The ethical principles of the World Medical Association and the Declaration of Helsinki [36] were followed in the organisation, conducting and reporting of this study. Informed consent was obtained from all individual members of Expert Panels 1 and 2. Permission to collect data from managers was obtained from all participating organisations in line with national standards and guidelines. A data protection notice and a prior data protection assessment of the study were prepared. An information letter was sent to informants by e‐mail, including contact details of the researchers, the subject and method of the study and information on the voluntary nature of participation. Informed consent was sought from participants when they responded to the online survey. The informants were informed about the anonymity and confidentiality of their responses. In reporting the results, the anonymity of informants was respected, meaning they cannot be identified as individuals.

## 5 Results

### 5.1 Forward Translation and Reconciliation

The Finnish translations by the two independent translators were very similar. There were minor differences in several items related to word choice, semantic content or word order. The study group evaluated T1 as being better on ten items while T2 was better on five items. The final version consisted of a combination of both translations, with seven items modified by the study group. For example, certain phrases and terms in the MEP‐Fin required rewording using alternative Finnish expressions. This process entailed prolonged deliberation and reflection within the research team to ensure conceptual and linguistic accuracy.

### 5.2 Back Translation Review and Harmonisation

The back translation was not identical to the original scale. However, in almost all cases, the items differed, for example, only in one word. Expert Panel 1 evaluated the back‐translated version of the scale as mostly matching the content of the original version. Finnish managers generally found all 24 items appropriate and necessary to describe the phenomena (Table S1). At the final stage, the scale developer analysed the latest version. After he approved the back translation without further modifications, the MEP‐Fin was ready for use in the cognitive debriefing interviews (Table S3).

### 5.3 Review of Cognitive Debriefing Results

The I‐CVI values after usability assessment were adequate for 20 out of 24 scale items. The MEP‐Fin showed high overall content validity (S‐CVI/Ave = 0.91). The mean score for the suitability and comprehensibility of the scale was 8.4. The individual items also scored high for content validity (I‐CVI range: 0.56–1.00), except for one (0.38), item 7, ‘Generating the greatest overall benefits for the district/hospital’ (Table 1).

**TABLE 1:** The item level content validity index (I‐CVI) values of the MEP‐fin from the assessment by expert panel 2.

Item	I‐CVI value of the MEP‐fin scale
1, 2, 3, 4, 5, 6, 8, 10, 13, 15, 16, 17, 18, 19, 20, 21, 22, 23, 24	1.0
7	0.38
11, 12, 14	0.56

Abbreviations: MEP‐fin, Finnish version of the MEP scale; The MEP scale, The Managerial Ethical Profile scale.

Necessary changes were made to the scale after the study group reviewed the evaluations of Expert Panel 2. Six items remained unchanged. The wording of 18 items was changed to make them more understandable. Some of these items were also reformulated to characterise them in more detail in line with the human element of health and social care management. The experts felt that existing items dealt with ethical decision‐making in health and social care management. However, a few culturally specific terms felt somewhat divergent in the Finnish management context. Some items caused confusion among experts in terms of overlapping content.

A minor change was made to item 1 with the word ‘providing’ being replaced with ‘achieving’ at the recommendation of the study group. This translation was found to be shorter and more direct. Items 4 and 7 stimulated debate and reflection; for example, regarding the back translation to the original English version of ‘district’, compared to the Finnish translation of ‘region’. These definitional differences were recognised. Managers and the study group felt the term ‘region’ was a better fit with Finnish managerial and geographical terminology than ‘district’.

Item 7, ‘Generating the greatest overall benefits for the district/hospital’ was also felt to be unclear and to overlap excessively with item 11, ‘Creating the greatest overall benefit for the local community’ and item 12, ‘Creating the greatest overall benefit for the wider community’. The experts suggested that those items were related and could be presented on the scale after each other.

Item 8 was complicated since ‘harm’ cannot be used as a verb in the Finnish language. Item 8, ‘Not harming the clients/patients’, was thus changed to ‘Avoiding causing harm to clients/patients’. Item 9, ‘Respecting organizational rules and regulations that have been created for the greatest benefit of all stakeholders’, required the addition of the adjective ‘possible’ to accurately describe ‘the greatest benefit’ in the Finnish language.

Variations in the organisation of health and social care in Finland required words to be changed in item 10, ‘Obeying the law (state and federal)’, to align with regulations. The terms ‘state and federal’ were not applicable since Finland has national laws and regulations. A few items were found to be lacking cultural relevance in Finland and to be linguistically different in Finnish. Item 13, ‘Being most in line with your core personal values’, was found to be too close to item 14, ‘Being most in line with the person you want to be’. Item 14 was evaluated as not being as relevant and important as item 13. The open‐ended comments also indicated that this item was not appropriate to Finnish culture. Item 15, ‘Respecting the dignity of those affected by the decision’, was changed to ‘Respecting the human dignity of the parties involved’ for linguistic and usability reasons.

Items 18, 21 and 24 needed modifications because there are no similar sayings or terms in the Finnish language. For this reason, the concepts underlying these items had to be clarified and explained in more detail. No conceptually problematic items were found that could not be overcome. Examples of the cognitive debriefing process and thought processes are presented in Tables S4 and S5. As this was the first attempt to test the instrument in the Finnish context, all original items were retained to allow for a comprehensive evaluation of its content validity.

### 5.4 Preliminary Psychometric Testing Results

Most of the participants who responded to the online survey were female, and their mean age was 51. Most of the participants had a bachelor's degree (82%) while the rest had a master's degree (18%). Three‐fourths of the participants worked as frontline managers (75%) and the rest worked in middle or top management positions. The participants had an average of around 11 years' work experience in their current positions. A high proportion (81%) of managers were engaged in management work full‐time (Table 2).

**TABLE 2:** Characteristics of the participants (*n* = 68).

Background information	*n* (%)	Mean (standard deviation)	Range
Gender			
Women	65 (95.6)		
Men	3 (4.4)		
Age			
< 50 years	27 (40)	51.2 (7.7)	31.0–65.0
≥ 50 years	41 (60)		
Educational level			
Bachelor level or lower	56 (82)		
Master level	12 (18)		
Working position			
Frontline manager^a^	51 (75)		
Middle/top manager^b^	17 (25)		
Years worked at current position			
< 5 years	22 (32)	10.9 (9.0)	0.3–35.5
≥ 5 years	46 (68)		
Time allocated to management work			
< 100%	13 (19)	93.1 (18.1)	(6.0–100.0)
100%	55 (81)		

Abbreviation: SD, standard deviation.

^a^ For example, Home care/service manager, service person, charge nurse.

^b^ For example, Head of outpatient services/division, director of basic services.

The following subscales had moderate correlations: reputational egoism with rule utilitarianism, rule utilitarianism with act utilitarianism and rule deontology, virtue of self with virtue of others and act deontology with rule deontology. The strongest correlation was between virtue of others and act deontology (Table 3). The internal consistency, as measured by Cronbach's alpha coefficient, of economic egoism (0.92), reputational egoism (0.75), rule utilitarianism (0.8), act utilitarianism (0.8) and virtue of self (0.93) subscales was good. However, the act deontology (0.58), virtue of others (0.54) and rule deontology (0.51) values dropped below an acceptable level in our study. The MEP‐Fin descriptive statistics and Cronbach's alpha values are presented in Table 4.

**TABLE 3:** Correlations for the subscales of the MEP‐fin (*n* = 68).

Subscales	1	2	3	4	5	6	7	8
Economic egoism	1							
2 Reputational egoism	0.25*	1						
3 Rule utilitarianism	0.18	0.56**	1					
4 Act utilitarianism	0.28*	0.36**	0.46**	1				
5 Virtue of self	0.11	0.20*	0.3	0.01	1			
6 Virtue of others	0.14	0.29*	0.37**	0.14	0.52**	1		
7 Act deontology	0.2	0.30**	0.38**	0.19	0.39**	0.74**	1	
8 Rule deontology	0.12	0.38**	0.54**	0.24*	0.23	0.63**	0.65**	1

*Note:* *p* value significant (two‐tailed) at level: Pearson's correlation coefficient, **p ≤ 0.01; *p ≤ 0.05.

Abbreviations: MEP‐fin, Finnish version of the MEP scale; The MEP scale, The Managerial Ethical Profile scale.

**TABLE 4:** Descriptive statistics and Cronbach's alphas of the Finnish MEP scale (*n* = 68).

Subscales	Number of items	Mean	SD	Cronbach's alpha
Economic egoism	3	3.24	0.67	0.92
2 Reputational egoism	2	3.99	0.82	0.75
3 Rule utilitarianism	2	4.47	0.61	0.8
4 Act utilitarianism	2	3.88	0.76	0.8
5 Virtue of self	2	4.1	0.93	0.93
6 Virtue of others	4	4.65	0.54	0.54
7 Act deontology	3	4.57	0.58	0.58
8 Rule deontology	3	4.78	0.51	0.51

*Note:* Range of scale, minimum‐maximum 1–5 SD, standard deviation.

Abbreviations: MEP‐fin, Finnish version of the MEP scale; The MEP scale, The Managerial Ethical Profile scale.

## 6 Discussion

To our knowledge this MEP‐Fin study is the first to translate and culturally adapt the MEP scale to a language other than English and a cultural area other than an Anglo‐Saxon region. In addition, we are not aware of other studies that have investigated ethical decision making in the integrated health and social care management context. Our study describes the creation of the MEP‐Fin using the ISPOR method, where cognitive interviews were combined with preliminary psychometric testing. This confirmed the quality, cultural adaptation and content validity of the translation of the MEP scale to the Finnish health and social care management context with the aim of preserving the original meaning of each item [26]. The translation, cultural adaptation and preliminary psychometric testing of the MEP‐Fin were performed by following a step‐by‐step process, including the recommended careful planning, preparation and precise methodological approaches that require time and expertise [27].

Our study process resulted in the MEP‐Fin, which is conceptually comparable to the original English version. The cultural adaptation based on the assessments of expert panels and supplemented by preliminary psychometric testing was rewarding. The items on the MEP‐Fin were found to be in line with the original mainly in terms of culture, values and supporting individual development as a manager. The translation and management expertise of participants complemented each other, thus avoiding misinterpretations. The MEP‐Fin includes items which were evaluated throughout this process by two distinct expert panels. An increase in the number of items may be necessary for some subscales, as now there are only two items on the original scale, representing reputational egoism, act utilitarianism and self‐virtue. Including health and social care managers in the process of cultural adaptation of the MEP scale was essential since they will be the final users of the MEP‐Fin, and they have deep experience of applying knowledge and making decisions in real day‐to‐day work.

The strongest correlation identified in our study was between the perceived virtue of others and act deontology, aligning with the findings reported by Casali (2011). In Casali's (2011) study, two virtue ethics subscales and two deontological subscales were strongly correlated, while in our study the correlations were moderate. For several subscales, the Cronbach's alpha values reported by Casali (2011) economic egoism (0.80), reputational egoism (0.69), rule utilitarianism (0.67) and act utilitarianism (0.88) were lower than those in our study. Meanwhile, Casali's (2010) virtue of self (0.84), virtue of others (0.72) and rule deontology (0.78) values were higher than those in our study. We tested the preliminary psychometric properties of the translated scale using conventional methods with people in the target group of interest, since this leads to a reliable and valid scale in the target language of interest [27].

Overall, the MEP‐Fin showed good coverage, content validity and internal consistency. However, the validity of the MEP‐Fin still requires further examination, including full psychometrics [27] such as confirmatory factor analysis and concurrent validity against another instrument.

Casali removed three items from the original 24‐item scale [15] (Table S1). These items should be tested in the Finnish context, as they were considered appropriate and necessary by Finnish managers and experts. Future studies could investigate the psychometric properties of the MEP‐Fin with a larger sample. Cultural adaptation, psychometric testing and validation of the MEP scale in other cultures are also recommended. More quantitative studies on EDM [6] are needed in general, especially in different health and social care management contexts and service systems [21]. Preliminary psychometric testing validated the MEP‐Fin, while further cross‐cultural studies are needed on EDM in general [4].

## 7 Strengths and Limitations

For the translation and cultural adaptation process we followed the steps of the ISPOR guidelines exactly. Besides that we conducted preliminary psychometric testing. The systematic adherence to the ISPOR protocol provided a strong methodological basis to ensure the quality and trustworthiness of the research.

The ISPOR protocol details very specific guidelines to ensure a scientific and transparent process. It was very important, however, that the original MEP scale developer provided support throughout the study process and explained the background and development of the different items in detail. Expert Panel 2, for its part, provided judgements on each item and acted as objective assessors. The scale developer accepted the back translation from Finnish to English without modifications. According to literature, accurate translation processes are likely to maintain the psychometric properties of the original source. The study reporting was based on a detailed description of the methodology used and all the specific steps concerning translation and cultural adaptation according to the COSMIN checklist.

The limitations of the translation processes were mainly related to the fact that the translators were not health or social care experts. The participants on the expert panels were not sufficiently diverse in terms of socio‐demographic characteristics, which should be considered when assessing the coverage of the sample and the generalisability of the results. Preliminary psychometric testing was performed at a later stage, not at the pre‐final stage and with a bilingual population as suggested. An online Webropol survey was chosen due to its broad geographical reach, cost‐effectiveness and convenience for participants. To increase the response rate, follow‐up messages were sent to the respondents twice every 2 weeks. Response bias was mitigated through rigorous attention to the entire online survey process, including neutral question design, pilot testing and systematic ordering of items. Participant engagement was enhanced through a cover letter emphasising the study's relevance and novelty. Socially desirable responses are always a potential concern and challenging to eliminate.

In this study, purposive sampling was employed, and we recommend that future studies further validate the MEP‐Fin in the Finnish health and social care sector with larger samples. Since the 30% response rate was relatively low, generalisation of results should proceed with caution.

## 8 Conclusion

The original 24‐item MEP scale was successfully translated and adapted to the Finnish health and social care context by following the ISPOR guidelines. Conceptual unity was attained in the translation. In the cultural adaptation phase, some of the items were redesigned based on the managers', experts' and study group's evaluations to make them more coherent and suitable for Finnish culture. The systematic translation, cultural adaptation and preliminary psychometric testing process resulted in a conceptually equivalent MEP‐Fin. It was found to be thorough, effective and relevant, and showed good coverage, content validity and internal consistency.

The MEP‐Fin includes items that comprehensively cover EDM characteristics and can be used to assess and guide ethically sustainable leadership in health and social care management in Finland. EDM is a core task and competence of managers in health and social care. The MEP‐Fin can be used for self‐evaluation, competence development and education of managers. Further pilot testing and evaluation of the psychometric properties of the adapted MEP‐Fin are needed to assess the acceptability and feasibility of using the questionnaire for managers in health and social care in Finland.

## Author Contributions

E.X., M.K., J.P. and O.K. were responsible for the study design. E.X. collected the data, E.X. and H.V. performed the data analysis, and E.X., M.K., J.P., H.V., N.M., L.C. and O.K. drafted the manuscript. All authors critically reviewed, analysed and approved the final version of this manuscript.

## Disclosure

Reporting Method: Reporting of the study adheres to the COnsensus‐based Standards for the selection of health status Measurement INstruments (COSMIN).

Permission to Reproduce Material From Other Sources: We hereby confirm that we have not used any materials from other researchers in this scientific article. All content, including text, figures and any other elements, is original and created by researchers.

## Ethics Statement

According to Finnish law and ethical guidelines, the research did not require approval from an ethics committee because it did not involve minors, direct or indirect physical or physiological harm to the participants or clinical trials.

## Conflicts of Interest

The authors declare no conflicts of interest.

## Supporting information


**Table S1:** The Original MEP (24/21 Items) and the Finnish MEP Scale.
**Table S2:** Definitions and Number of the Items in the MEP Scales Subscales.
**Table S3:** Examples of Differences Between the Original and Back Translated Versions of the MEP Scale.
**Table S4:** Examples of Item Modifications.
**Table S5:** Examples of Thought Processes in Cognitive Interviews.

## Data Availability

Survey data from the online survey will not be shared. The results of the translation and cultural adaptation assessment are presented in the Supporting Information.
