# Supplementary material for: Translation, Cultural Adaptation and Preliminary Psychometric Testing of the Managerial Ethical Profile Scale for Finnish Health and Social Care Sector
Source: Scand J Caring Sci. 2025 Aug 31;39(3):e70101. doi: 10.1111/scs.70101 (PMC12399779; doi:10.1111/scs.70101)
Supplement: Supplementary file 1 — Table S1: The Original MEP (24/21 Items) and the Finnish MEP Scale. Table S2: Definitions and Number of the Items in the MEP Scales Subscales. Table S3: Examples of Differences Between the Original and Back Translated Versions of the MEP Scale. Table S4: Examples of Item Modifications. Table S5: Examples of Thought Processes in Cognitive Interviews. [file SCS-39-0-s001.docx]

**Table S1 THE ORIGINAL MEP (24-/21-ITEMS) AND THE FINNISH MEP SCALE**

Merkitse, kuinka tärkeitä seuraavat asiat ovat päätöksenteossasi silloin, kun toimit organisaatiossasi

toimenkuvasi mukaisissa tehtävissä

(Please indicate how important the following issues are to your decision-making when you are working

in your organization in your job role)

Asteikko (Scale): Ei lainkaan tärkeää- Ei kovin tärkeää- Melko tärkeää- Hyvin tärkeää-Erittäin tärkeää

(Very Important-Very Important-Somewhat important-Not very important-Not important at all)

Table S1 the original MEP scale (24-/21-items) and the MEP-Fin

| THE ORIGINAL MEP SCALE STATEMENT | THE FINNISH MEP SCALE STATEMENT |
| --- | --- |
| 1.Providing the highest economic at return (profit) for the organization | 1.Mahdollisimman suuren taloudellisen voiton tuottaminen organisaatiolle |
| 2. Minimizing costs for the organization | 2.Organisaatiolle aiheutuvien kustannusten minimointi |
| 3.Protecting the reputation of the organization | 3.Organisaationi maineen ylläpitäminen |
| 4. Optimizing resources of the at district/hospital/unit/dept | 4. Alueen/sairaalan/yksikön/osaston resurssien optimoiminen |
| 5. Attaining organizational yearly at budgets (short term) * | 5.Organisaation vuosittaisessa budjetissa pysyminen* |
| 6.Being in line with the organizational mission | 6. Organisaation mission mukaisesti toimiminen |
| 7. Generating the greatest overall benefits for district/hospital* | 7. Suurimman mahdollisen hyödyn saavuttaminen alueelle/sairaalalle* |
| 8. Not harming the clients/patients | 8. Asiakkaille/potilaille koituvien haittojen välttäminen |
| 9. Respecting organizational' rules and regulations that have been created for the greatest benefit for all stakeholders | 9. Kunnioitetaan organisaation sääntöjä ja määräyksiä, jotka ovat laadittu mahdollisimman suuren hyödyn tuottamiseksi sidosryhmille |
| 10. Obeying the law (state and federal) * | 10. Lakien ja asetusten noudattaminen * |
| 11. Creating the greatest overall benefit for the local community | 11. Suurimman kokonaishyödyn tuottaminen paikallisesti |
| 12. Creating the greatest overall benefit for the wider community | 12. Suurimman kokonaishyödyn tuottaminen laajemmalle yhteisölle |
| 13. Being most in line with your core personal values | 13. Toiminen mahdollisuuksien mukaan henkilökohtaisten arvojesi mukaisesti |
| 14. Being most in line with the person you want to be | 14. Toimiminen mahdollisuuksien mukaan sen mukaisesti, millainen ihminen haluat olla |
| 15. Respecting dignity of those affected by the decision | 15. Asianosaisten ihmisarvon kunnioittaminen |
| 16. Being able to empathize with clients | 16. Kyky kokea empatiaa asiakkaita kohtaan |
| 17. Acting openly when making decision | 17. Avoimesti toimiminen päätöstä tehdessä |
| 18. Making "care for the sick" paramount | 18. Haavoittuvassa asemassa olevien asettaminen etusijalle |
| 19. Giving the opportunity to all affected at parties or their representatives to have input into the decision-making process | 19. Kaikkien asianosaisten tai teidän edustajiensa osallistuminen päätöksentekoon |
| 20. Treating others as you want others to treat you | 20. Muiden kohteleminen samoin kuin toivot itseäsi kohdeltavan |
| 21. Treat people as ends not as means | 21. Ihmisten arvokas kohtelu (ei vain välineinä tavoitteiden saavuttamiseen) |
| 22. Ensuring that confidentiality is maintained at all times | 22. Luottamuksellisuuden varmistaminen kaikissa tilanteissa |
| 23. Maintaining a fair process at all times | 23. Prosessien oikeudenmukaisuuden varmistaminen kaikissa tilanteissa |
| 24. Ensuring that the organization "duty of care" is maintained at all times | 24. Asiakkaan aseman ja oikeuksien huomioiminen kaikissa tilanteissa |

The MEP scale= The Managerial Ethical Profile scale

MEP-Fin=Finnish version of the MEP scale

*Dropped from Casali’s the best fit 21-item model

**Table S2 DEFINITIONS AND NUMBER OF THE ITEMS IN THE MEP SCALES SUBSCALES**

Table S2. Definitions and number of the items in the MEP scales subscales according to Casali (2010)

| Subscale | Definition | Number of the item |
| --- | --- | --- |
| Economic egoism | Managers self-interest in economical outcomes (e.g. profit and cost reduction). | 4 |
| Reputational egoism | Introduces the managers identification process, “organization as an extension of one’s own interests” by protecting the reputation even at the expense of profits. | 2 |
| Act utilitarianism | A process where to create overall maximal good it is necessary to evaluate whether the consequences of planned action will provide the greatest benefit for the significant number of stakeholders. | 2 |
| Rule utilitarianism | Searches also greater good but not from single actions but it proposes to make rules and to follow them so that the majority can benefit the most. | 3 |
| Self-virtue | Good decisions which are made by people who exhibit good personal character virtues. | 2 |
| Others virtue | Living well with others, promoting good social wellbeing and which includes care ethics. | 4 |
| Act deontology | The notion that the goodness of the act is not determined by inhuman application of a moral principle, but by completing what duty is needed in each situation. | 3 |
| Rule deontology | Fulfilling universal duties, similarly as the Golden rule, or acting according to universal fundamentals (e.g. justice, not harming others, doing good and respecting autonomy) in all situations. | 4 |

The MEP scale= The Managerial Ethical Profile scale

**Table S3. EXAMPLES OF DIFFERENCES BETWEEN THE ORIGINAL AND BACK TRANSLATED VERSIONS OF THE MEP SCALE**

Table S3. Examples of differences between the original and back translated versions of the MEP scale, as evaluating by expert panel 1

| Original  MEP SCALE | Back-translated version | Expert evaluation and discussion during harmonization | The Final back translation |
| --- | --- | --- | --- |
| Optimizing resources in the district/hospital/unit/department | Optimising resources in the region/hospital/unit/ward/department | Slightly different item  *Region* is a more appropriate term in this context | Optimising resources in the region/hospital/unit/ward/department |
| Being in line with the organizational mission | Acting according to the organization’s mission statement | Significant difference  *Being in line* is more common in practice, *statement* restrictive term | Being in line with the organizational mission |
| Obeying the law (state and federal) | Complying with laws and regulations | Slightly different item  Finnish legislation is based on laws and *regulations* | Obeying the law and regulations |
| Creating the greatest overall benefit for the local community | Producing the greatest possible benefit for the local community | Slightly different item  The adjective “greatest possible” describes the best way in the Finnish language the greatest benefit. | Producing the greatest possible benefit for the local community |
| Making "care for the sick" paramount | Making people who are in a vulnerable position the priority when specifying options | Significant difference  There is not similar saying or term in the Finnish language | Making people who are in a vulnerable position the priority when specifying options |

MEP scale= Managerial ethical profile

**Table S4. EXAMPLES OF ITEM MODIFICATIONS**

Table S4. Examples of item modifications based on the review of cognitive debriefing I results

| The item before cognitive debriefing I | Comments from cognitive debriefing I | Reasons for  changes in the item based on cognitive debriefing I results | The item after cognitive debriefing I results |
| --- | --- | --- | --- |
| Organisaation toiminta-ajatuksen mukaisesti toimiminen  (Acting in accordance with the organizations’ mission statement) | “Would the operating principle be a better term?”  *The term mission here?* | Item reworded to be more familiar in organizational practice and using a broader/ not so limited concept | Organisaation mission mukaisesti toimiminen  (Being in line with the organizational mission) |
| Asiakkaille/potilaille koituvien vahinkojen välttäminen  (Not harming the clients/patients) | *“Could the word be a harm, not an injury?”*  *“I wonder if a more neutral one might be more appropriate for those caused by but resulting from”* | Item reworded to be more understandable and consistent in Finnish | Asiakkaille/potilaille koituvien haittojen välttäminen  (Avoiding harm to clients/patients) |
| Kyky empatiaan suhteessa asiakkaisiin  (Being able to empathize with clients) | *“Ability to empathize with clients (should have better Finnish)”*  *“Or empathic encounter?”* | Item reworded making the item more precise and suitable for the Finnish health and social care culture and philosophy | Kyky kokea empatiaa asiakkaita kohtaan  (Ability to empathise with clients) |

MEP scale= Managerial ethical profile

**Table S5. EXAMPLES OF THOUGHT PROCESSES IN COGNITIVE INTERVIEWS**

Table S5. Examples of thought processes in cognitive interviews

| Item | Thought process |
| --- | --- |
| Organisaation maineen suojeleminen  (Protecting the reputation of the organization) | *“An important question, but the choice of the word "protect" suggests that there is something to hide. Maintaining?”*  *“Essentially related to ethics, confidentiality”*  *“Here's an assumption that the reputation is already good”* |
| Ihmisten arvokas kohtelu, ei vain välineinä tavoitteiden saavuttamiseen  (Treat people as ends not as means) | *“Important topic, this content does not reach”*  *“Does this mean "putting the client at the centre"*  *“A cultural issue”* |

MEP scale= Managerial ethical profile
